# Notes and News

**Published:** 1905-09-30

**Authors:** 


### Notes and News.

Yellow Fever is very prevalent in New Orleans.

The members of Lloyd's have contributed £2,584 toward the Guy's Hospital Appeal Fund.

Professor Crothers, M.D., will deliver the first Norman Kerr lecture before the Society for the Study of Inebriety on October 10th, at 8.30, in the rooms of the Royal Medical and Chirurgical Society.

A case of cholera is reported in Berlin, where the most strenuous measures are being taken to prevent an outbreak. Both Russia and France are taking precautions to prevent the epidemic being introduced from Prussia. Five deaths are reported from Lodz, and one at Bloclawek in the Government of Warsaw.

The lectures and discussions arranged by the Childhood Society and British Child-study Association, at the Parkes Museum, will commence on October 19th. The Hon. Sir John Cockburn, M.D., Cecil Reddie, Ph.D., Professor Earl Barnes, Dr. M. Friendeberger, Professor Halleburton, M.D., are amongst those who will lecture.

A very excellent suggestion was made the other day relative to poison bottles. This was that each poison should bear on its label the name of its particular antidote or antidotes. This suggestion may be adopted at once, by those who have poisons in their possession, and no doubt the trade will finally adopt the suggestion, which might well be made compulsory.

Professor Fuchs, of Friburg, who visited Liverpool on behalf of the German Union for Encouraging Workmen's Houses, paid a great compliment to the city. He said that in Liverpool they had established an ideal which he hoped German housing reformers would strive after. Whilst other municipalities had pulled down slums, they had not succeeded as Liverpool had; in rehousing the people Liverpool has spent about £900,000 in this direction.

Over 200 delegates attended Congress of the International Surgical Society at Brussels.

The annual British Homœopathic Congress was held at Hastings on Friday last. The next Congress is to be in London.

A new infirmary for females is to be erected for the Knaresborough Union. It will contain accommodation for 60 patients.

Convalescent wards are about to be added to the Grimsby Hospital through contributions from the Grimsby Amalgamated Friendly Societies.

Dr. F. J. Waldo, barrister of the Middle Temple, coroner for the City of London and Southwark, has been placed on the Commission of the Peace for the county of London.

The prizes to the students of the Royal Dental Hospital will be presented by Professor Osler on October 13, at 8 o'clock, and at 8.30 a conversazione will be held.

A new wing has been opened at the Devon and Cornwall Sanatorium, Didworth. This will add 18 beds to the institution at a cost of £800 for building and a further sum for furnishing and fitting.

Mr. Henry Rundle, a member of the Senior Surgical Staff of the Royal Portsmouth Hospital, has offered to erect and furnish a room to be used as a museum and medical reference library, in connection with the new operation theatre, the foundation-stone of which will probably be laid in the latter part of October by the Mayor of Portsmouth.

A sanatorium and farm colony for 64 children is to be erected at Stannington, Northumberland, by the Newcastle and Gateshead Poor Children's Rescue Agency. The scheme has been possible through the generosity of Mr. Roland Philipson of Newcastle. There will also be a convalescent home for 60 boys.

The Harben Lectures will be delivered in the Lecture Room of the Royal Institute of Public Health on October 10th, 12th, and 17th, at 5 p.m. by Professor Thomas Oliver, M.A., M.D., LL.D., F.R.C.P., physician to the Royal Infirmary, Newcastle-on-Tyne. Subject: "Some of the Maladies caused by the air we breathe in the Home, the Factory, and the Mine, including a description of Caisson Disease or Compressed Air Illness." The lectures are open to the public.

The late Mrs. Irene Forrest of Lytham has bequeathed £1,000 to the Lytham Cottage Hospital. The late Mrs. M. H. Hawtrey of South Kensington has made the North-Eastern Hospital for Children residuary legatee of £6,000. The late Mr. R. B. Blaize of Lagos has left provisions for the erection of a hospital at Abeo Ruta for the natives and other poor. He has also arranged that his son is eventually to take charge of the hospital, where he is to remain for three years. Afterwards a hospital is to be built at Oyo, which his son is then to superintend.

